# No effect on pharmacokinetics of tamoxifen and 4-hydroxytamoxifen by multiple doses of red clover capsule in rats

**DOI:** 10.1038/srep16126

**Published:** 2015-11-04

**Authors:** Kanumuri Siva Rama Raju, Isha Taneja, Guru Raghavendra Valicherla, Murali Krishna Challagundla, Mamunur Rashid, Anees Ahmed Syed, Jiaur Rahman Gayen, Sheelendra Pratap Singh, Muhammad Wahajuddin

**Affiliations:** 1 Academy of Scientific and Innovative Research, New Delhi, India; 2 Pharmacokinetics and Metabolism Division, CSIR- Central Drug Research Institute, Lucknow-226031, India; 3 Analytical Chemistry Lab, CSIR-Indian Institute of Toxicology Research, Lucknow-226001, India; 4 Department of Pharmaceutics, National Institute of Pharmaceutical Education and Research, Raebareli-229010.

## Abstract

Tamoxifen is used in clinical practice for breast cancer patients and to prevent osteoporosis. Red clover (*Trifolium pratense*) preparations are consumed worldwide as dietary supplements for relieving postmenopausal symptoms. In the present study we investigated the possible herb-drug interaction between red clover and tamoxifen in rats. 15 days pre-treatment with red clover did not alter the tamoxifen and its active metabolite 4-hydroxytamoxifen pharmacokinetics significantly (p > 0.05). Therefore the therapeutic efficacy of the tamoxifen may not be compromised by the co-administration with red clover. Tamoxifen metabolism is primarily mediated by CYP2D6, CYP3A4 with minor contribution from CYP2C9, CYP2E1 and CYP1A2 isoforms. Although, red clover pre-treatment significantly (p < 0.05) decreased the mRNA expression and activity of CYP3a2, no effect on CYP2d4 and increased expression and activity of CYP2c11 could be the plausible reasons for lack of effect on tamoxifen and its metabolite pharmacokinetics in rats. CYP1a1 and CYP2b2 mRNA expression and activity were also significantly reduced by red clover. To extend the clinical utility of the present study, effect of red clover extract on major CYPs using human liver microsomes and HepG2 cell lines were also determined. Similar finding were observed in the human liver preparations as in rats.

*Trifolium pratense*, commonly known as red clover, is a medicinal herb, traditionally used for the treatment of chronic skin diseases and whooping cough. Biochainin A (BCA), formononetin (FMN), daidzein (DZN) and genstein (GEN) are the four principal phytoestrogens in red clover preparations, such as Promensil^®^ (Novogen Inc., Samford, CT, USA) that are marketed as dietary supplements for relieving postmenopausal symptoms such as hot flashes, bone loss, breast cancer and for maintaining men’s prostate health. In various *in vitro* and *in vivo* studies, the principal constituents of red clover have been shown to be potent inhibitor of the efflux transporters viz. P-glycoprotein (P-gp) and breast cancer resistance protein (BCRP), and CYP450 mediated metabolism^1^,^2^,^3^,^4^. It is evident that biochanin A, one of the major constituent of red clover has been reported to have modulating effects on pharmacokinetics of several drugs such as tamoxifen, paclitaxel, digoxin, fexofenadine and mitoxantrone^5^,^6^,^7^,^8^,^9^. Other two important constituents of red clover, daidzein and genistein are also well-known modulators of CYP1A1, 1B1, 1A6, SULTs, UGTs and transporters affecting the bioavailability of tamoxifen, theophylline, oestradiol, DHEA, 4-methyl umbelliferone, nicotine, paclitaxel, daunomycin, vinblastine, mitoxantrone and nitrofurantoin^5^,^10^,^11^,^12^,^13^,^14^,^15^,^16^,^17^,^18^,^19^,^20^,^21^,^22^. All these studies have been conducted with the individual pure constituents and may not represent the true outcome of the red clover consumption as such, which contains mixture of several isoflavonoids that could have synergistic or antagonistic properties when given together. Further, these studies were conducted at higher doses which may not be achieved when ingested in the extract form. This necessitates the need to explore the herb-drug interactions of the red clover extract. The effect of red clover extract on the major CYP enzymes was recently published by our lab but it limits only to its effects on the expression level of enzymes up on treatment for one week^23^.

Tamoxifen, the prototypical selective estrogen receptor modulators (SERM), is used clinically in breast cancer patients. It acts as an estrogen antagonist in breast tissue, slowing cancer cell proliferation and an estrogen agonist in bone tissue and in cardiovascular system to prevent osteoporosis and heart diseases, respectively. Oral tamoxifen undergoes extensive hepatic metabolism and subsequent biliary excretion of its metabolites. The main pathway for tamoxifen biotransformation is via its hydroxylation to form 4-hydroxy tamoxifen, catalyzed primarily by CYP2D6^24^,^25^,^26^. Among the serum metabolites of tamoxifen, 4-hydroxytamoxifen has received particular attention since it is 30 to 100 times more potent than the parent drug as an estrogen antagonist because of its higher *in vitro* affinity towards the estrogen receptor than the parent drug^27^,^28^. Tamoxifen and its active metabolite 4-hydroxytamoxifen are substrates of P-gp, BCRP and multidrug resistance-associated protein (MRP) 2^29^,^30^. The chances of co-administration of tamoxifen and red clover are very likely in breast cancer and postmenopausal women. Being a substrate of transporters and extensive CYP450 mediated metabolism makes tamoxifen vulnerable for interactions with red clover. Previous reports show that the major metabolites obtained in human liver microsomes resemble qualitatively with that obtained in rat liver microsomes^31^,^32^. Therefore, in the present study we investigated the effect of chronic administration of marketed red clover preparation on pharmacokinetics of tamoxifen in rats for the first time. The amounts of major isoflavonoids present in the selected red clover extract are determined for the purpose of standardization. We also studied the effect of marketed red clover preparation on the major CYP enzymes responsible for metabolism of tamoxifen in terms of both expression at mRNA level and microsomal activity after the chronic treatment for 15 days in rats to explore the possible reasons that could explain the conceivable interactions. We also determined the effect of red clover extract on the activity of CYPs in human liver microsomes and possible induction in HepG2 cells upon 3-day treatment.

## Results

### Analysis of red clover capsule content

Upon HPLC analysis of the red clover capsule content, the isoflavonoids formononetin, biochanin A, genstein and daidzein were found be present in the concentrations of 1.59, 2.03, 0.94 and 1.36 mg/g of capsule content.

### Effect of red clover pre-treatment on the pharmacokinetics of tamoxifen

**Figure 1:**
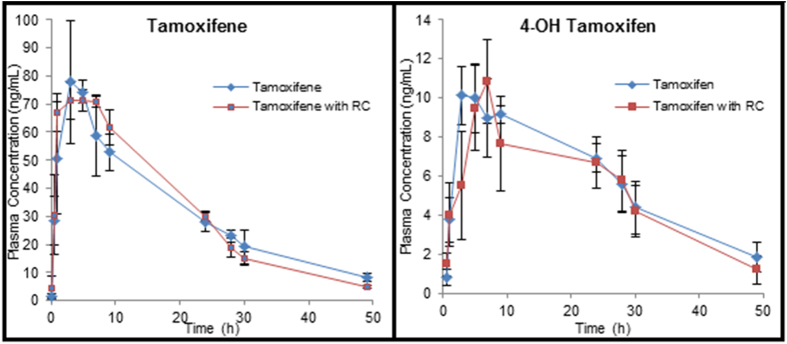
Mean plasma concentration–time profiles of tamoxifen and 4-hydroxytamoxifen after the oral administration of tamoxifen (10 mg/kg) with or without red clover (45 mg/kg/day) to rats. Bars represent the standard deviation (n = 5).

The mean plasma concentration–time profiles of tamoxifen and its active metabolite, 4-hydroxy tamoxifen, upon tamoxifen administration (10 mg/kg) alone or in combination with red clover extract (45 mg/kg/day) orally in rats, are shown in Fig. 1, while the pharmacokinetic parameters are summarized in Table 1. Pretreatment with red clover for two weeks did not have any significant effect on the peak plasma concentration and the AUC_0-∞_ of tamoxifen (Table 1). There was no significant change in the peak plasma concentration and the AUC_0-∞_ of 4-hydroxy tamoxifen also (Table 1). Upon red clover pre-treatment, the relative bioavailabity (RB%) and mean metabolite ratio (MR) of tamoxifen were reduced from 100 and 0.20 to 96.85% and 0.18, respectively, which was not significant (p > 0.05).

### Effect of red clover treatment on CYP enzyme expression

**Figure 2:**
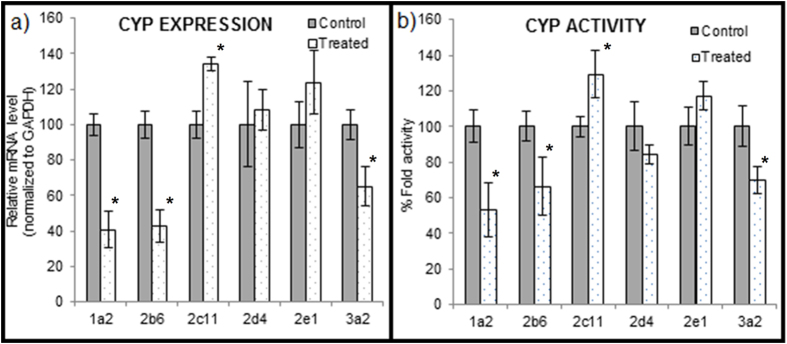
The effect of red clover treatment for 15 days on the expression levels and activities of the CYP enzymes 1a1, 2b2, 2c11, 2d4, 2e1 and 3a2 with respect to the rats administered with vehicle for 15 days (n = 5). (*represents the p < 0.05, significantly different from control).

The effect of red clover treatment on expression of CYP enzymes like CYP1a1, CYP2b2, CYP2c11, CYP2d4, CYP2e1 and CYP3a2 are shown in Fig. 2. The expression of CYP1a1, CYP2b2 and CYP3a2 were significantly decreased to 40.88 ± 10.44%, 42.79 ± 9.36% and 65.38 ± 11.23%, respectively while the expression levels of CYP2c11 was significantly increase to 134.39 ± 3.82% as compared to control rats. There was no significant effect of chronic treatment of red clover on CYP2d4 and CYP2e1 expression, which were found to be 108.38 ± 11.49% and 123.71 ± 18.02%, respectively as compared to control rats.

### Effect of red clover on the CYP isozymes activity

We determined the effect of red clover on the activity of major CYP enzymes which are involved in metabolism of many drug substances. The enzyme activities of the red clover treated rat liver microsomes to that of the vehicle treated rats to assess the effect of red clover treatment on CYP enzymes activity. The activities of the CYP 1a1, 2b2 and 3a2 were decreased significantly to 53.34 ± 14.99%, 66.28 ± 16.28% and 70.03 ± 7.66% respectively up on pre-treatment with red clover extract (Fig. 2). The activities of 2d4 and 2e1 were not altered significantly while the activity of 2c11 increased significantly to 129.31 ± 13.54% compared to vehicle treatment group.

### Estimation of IC_50_s in human liver microsomes

The inhibitory potential of the red clover extract on the major CYPs in human liver microsomes expressed as IC_50_ are as shown in Table 2. Red clover extract inhibited conversion of phenacetin to acetaminophen catalysed by CYP1A2 in a concentration dependent manner with IC_50_ of 80.91 μg/mL. CYP3A4 activity was also inhibited by red clover extract (IC_50_ = 89.52 μg/mL) while it moderately inhibited the activity of CYP2C9 (IC_50_ = 141.2 μg/mL). There were weak or inconspicuous effects of red clover on the catalytic efficiency of other CYP isoforms studied within the concentration range tested.

### Effect of red clover treatment on CYP enzyme expression in HepG2 cells

**Figure 3:**
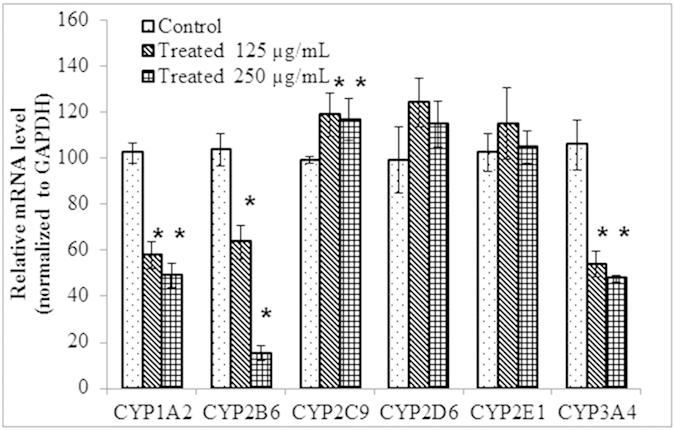
The effect of red clover treatment for 3 days on the expression levels of the CYP enzymes 1A2, 2B6, 2C9, 2D6, 2E1 and 3A4 with respect to the control group (n = 6). (*represents the p < 0.05, significantly different from control).

The effect of red clover treatment on expression of CYP enzymes CYP1A2, CYP2B6, CYP2C9, CYP2D6, CYP2E1, CYP2B6 and CYP3A4 in HepG2 cells are shown in Fig. 3. The expression of CYP1A2, CYP2B6 and CYP3A4 were significantly decreased in a concentration dependent manner (Fig. 3) while the expression level of CYP2C9 was significantly increased as compared to control group. There was no significant effect of chronic treatment of red clover extract on CYP2D6 and CYP2E1 expression, as compared to control group.

## Discussion

Due to worldwide rise in the use of herbal preparations, more preclinical or clinical data regarding herb-drug pharmacokinetic interactions is needed to make informed decisions regarding patient safety. Red clover is available in market in different preparations such as teas, tinctures, capsules, tablets, liquid extracts, extracts standardized for specific isoflavone content, ointment etc. for relieving postmenopausal symptoms such as hot flashes, bone loss, breast cancer and for maintaining men’s prostate health. Tamoxifen is the most commonly used SERM in clinical practice for breast cancer patients and for prevention of osteoporosis. Therefore, the co-administration of tamoxifen with red clover preparations is very likely in this population group. However, no reports are available till date stating the effect of red clover on the pharmacokinetics of tamoxifen. Therefore, in the present study we investigated the effect of chronic administration of red clover preparation on pharmacokinetics of tamoxifen in rats. The results of this study suggests that the chronic pretreatment of red clover does not alter the pharmacokinetics of tamoxifen or the systemic exposure of the active metabolite, 4-hydroxy tamoxifen significantly (p > 0.05) (Table 1). To understand the possible reasons for the absence of significant interaction of red clover extract with tamoxifen, we explored the effect red clover pre-treatment on the major CYP enzymes responsible for the metabolism of several marketed drugs and especially tamoxifen. We studied the enzyme expression levels and activities of the red clover treated rat liver microsomes in comparison to the vehicle treated rats to assess the effect of red clover treatment on major CYP isoforms. In red clover treated rat liver microsomes, the mRNA expression level of CYP1a1, 2b2 and CYP3a2 was significantly lowered to 40.88, 42.79 and 65.38% respectively, while CYP2c11 was significantly increased to 134.39% (p < 0.05). Similarly, the activities of the CYP 1a1, 2b2 and 3a2 were decreased significantly to 53.34, 66.28 and 70.03% respectively, while 2c11 increased significantly to 129.31% upon pre-treatment with red clover extract (p < 0.05). The expression levels and activities of 2d4 and 2e1 were not significantly altered. These results clearly indicate the possibility of the interaction of red clover with the drugs that are substrates of CYP 1a1, 2b2, 3a2 and 2c11.

In humans, tamoxifen metabolism occurs primarily via two pathways, hydroxylation and Ndemethylation to form 4-hydroxy tamoxifen and N-desmethyl tamoxifen, respectively. Of these, hydroxylation has been investigated rigorously since the resultant metabolite, 4-hydroxy tamoxifen is 30–100 times more active than the parent^27^,^28^. However, this pathway accounts for only 7% of the total metabolism of tamoxifen and is primarily mediated by CYP2D6, with minor contribution from CYP 2C9, 2B6, 3A4 and 2E1. The other pathway Ndemethylation to form N-desmethyl tamoxifen accounts for about 92% of the metabolism, which is primarily mediated by CYP 3A4 with the involvement of CYP 2D6, 2C9 and 1A2^33^,^34^,^35^,^36^.

Therefore, the lack of effect of red clover treatment on the systemic exposure of the 4-hydroxy tamoxifen in rats could be due to the absence of the inhibition or induction of CYP2d4. Although, the second major metabolic pathway of tamoxifen (N-demethylation) is mediated primarily by CYP3a4, it is also mediated by other CYP enzymes like 2d4 whose levels were unaltered and 2c11 which was induced by red clover treatment. These enzymes may be compensating the metabolism of the tamoxifen to N-desmethyl tamoxifen as the systemic levels of the tamoxifen that can be achieved are far below the k_m_ value of the tamoxifen metabolism to N-desmethyl tamoxifen in rats^37^.

Further to address the effect of the red clover extract on the microsomal CYP activities in human, its inhibitory potential (IC_50_ values) on CYPs were determined in human liver microsomes. The inhibition was found to be significant on CYP1A2, 3A4 and 2B6 with IC_50_ values of 80.91, 89.52 and 141.20 μg/mL, respectively. The inhibition of other isozymes CYP 2C9, 2D6 and 2E1 were negligible. To further assess the probability of *in vivo* herb-drug interaction potential of red clover constituents, we compared the maximum concentration of major isoflavones that can be achievable systemically (C_max_) and their IC_50_ values. The total systemic exposure of the isoflavones upon Promensil^®^ administration daily twice for 2 weeks was reported to be around 236.04 ng/mL^38^. The IC_50_ value of total major four isoflavone content obtained were 478.98 and 529.96 μg/mL for CYP1A2 and 3A4 respectively. It clearly indicates that there are negligible chances of interaction of the CYP substrates upon co-administration with red clover.

Further, to study the inductive effects of red clover on human CYPs at molecular level, the effect of 3-day red clover treatment on the expression levels of major CYPs in the HepG2 cells was determined. The expression levels of CYP 1A2, 2B6 and 3A4 were decreased significantly (p < 0.05) in a concentration dependent manner upon red clover extract treatment (Fig. 3). The mRNA expression of the 2C9 was increased significantly (p < 0.05) upon treatment with red clover extract. These results are in agreement with those observed in rats upon chronic administration. There was no effect on the expression of 2D6 and 2E1.

Though the administration of pure constituents of red clover is well known to alter the pharmacokinetics of several drugs^5^,^7^,^8^,^11^,^17^,^20^,^23^; in present study we could not find any significant interaction of red clover extract with tamoxifen. The reason for this insignificant interaction may be the low concentration of these isoflavones in red clover extract. Based on the HPLC analysis of the extract, the doses of the isoflavones formononetin, biochanin A, genstein and daidzein administered to the rats were 0.07, 0.09, 0.04 and 0.06 mg/kg, respectively when the 45 mg/kg/day dose of red clover extract was given to the rats. These dose doses are far below the tested doses of the red clover isoflavonoids tested for their pharmacokinetic interaction potentials. Therefore, upon red clover administration, there could be low circulating concentrations of these isoflavones as compared to when they are given as pure isoflavones at higher doses. This could result in lack of the potential to modulate the drug metabolizing enzymes at such low levels. These results indicate that there are very few chances of having interaction with co-administered tamoxifen in both humans and rats.

## Materials and Methods

### Chemicals and reagents

**Figure 4:**
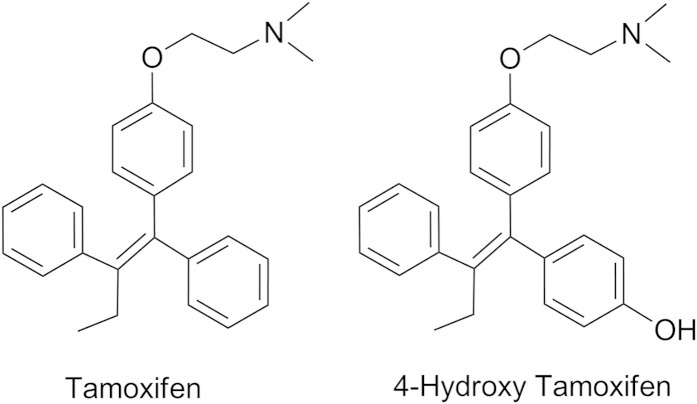
The chemical structures of tamoxifen and 4-hydroxy tamoxifen.

Tamoxifen and 4-hydroxytamoxifen were purchased from Sigma Aldrich Ltd (St Louis, USA). The chemical structures of the tamoxifen and 4-hydroxy tamoxifen are as shown in Fig. 4. Red clover capsules were purchased from pharmacy store in Canada (Red Clover Blossoms Capsules, Holland & Barrett Limited, Dublin). Centchroman (Internal standard, IS) was synthesized at the Medicinal Chemistry Division of Central Drug Research Institute (CDRI, Lucknow, India). Testosterone, 6-hydroxy testosterone, phenacetin, paracetamol, bupropion, hydroxy bupropion, diclofenac, 4-hydroxy diclofenac, dextromethorphan, chlorzoxazone and 6-hydroxy chlorzoxazone were purchased from Sigma Aldrich Ltd (St Louis, USA). HPLC grade acetonitrile, methanol and isopropanol were purchased from Sisco Research Laboratories (SRL) Pvt. Limited (Mumbai, India). HPLC grade n-hexane, ammonium acetate, isopropanol and glacial acetic acid AR were purchased from E Merck Limited (Mumbai, India). Milli-Q pure water was obtained from a Millipore Elix water purification system purchased from Millipore India Pvt. Ltd. (New Delhi, India). Sodium carboxy methyl cellulose (CMC) was purchased from Sigma Aldrich Ltd (St Louis, USA). Heparin sodium injection I.P. (1000 IU/mL) was purchased from Gland Pharma (Hyderabad, India). TRIzol reagent, Fetal Bovine Serum (FBS), Trypsin-EDTA and Dulbecco’s Modified Eagle’s Medium (DMEM) were purchased from Sigma Aldrich, India. Antibiotic-antimycotic solution and high capacity RNA to cDNA Reverse Transcriptase kit was purchased from Applied Biosystems, USA. 2X SYBR green Premix Ex Taq was purchased from Takara Bio, Japan. Primers were designed online using http://www.roche-applied-science.com and supplied by Eurofins genomics (Bangalore, India) and all the primers were high purity salt free (HPSF) purified (Table 3). All other chemicals and reagents were of analytical grade.

### Preparation of Red clover extract for the *in vitro* studies

The red clover capsules used for the study were characterized for their major isoflavone content^39^. The contents of 10 Red clover capsules were pooled and 500 mg of the powder was taken in a 50 ml conical flask and 30 ml mixture of methanol: triple distilled water 80:20% (v/v) containing 0.5% ortho-phosphoric acid was added, kept under shaking for 2 h and then sonicated for 30 min. Then the volume was made upto 50 mL. The mixture was centrifuged at 5000 g and the supernatant was evaporated to dryness using nitrogen gas drier at 45 °C under vacuum. The residue was then reconstituted in 1 mL of the methanol and used for further studies. The extract was diluted to 1 mg/mL with methanol and analyzed by HPLC-Photo Diode Array using Waters XBridge^®^ C18 column (250 × 4.5 mm, 5 μm). A gradient program with 0.1% formic acid (A) and Acetonitrile with 0.1% formic acid (B) in linear gradient from 20% to 40% A in 40 min and re-equilibration in 2 min was run. The chromatogram was monitored at 250 nm wavelength.

### Animals

Young, adult female *Sprague–Dawley* rats, weighing 200–220 g, were procured from the National Laboratory Animal Center, CDRI (Lucknow, India). Rats were housed in well ventilated cages at room temperature (24 ± 2 °C) and 40–60% relative humidity while on a regular 12 h light-dark cycle. The animals were acclimatized for a minimum period of one week prior to the experiment. Approval for animal experimentation from the Local Animal Ethics Committee of CSIR-Central Drug Research Institute was sought and all the animal studies were carried out in accordance with the approved guidelines and regulations.

### Pharmacokinetic study

The rats were fasted overnight (14–16 h) prior to the experiment but given free access to water. Tamoxifen solution was prepared by dissolving it in 0.9% NaCl-injectable solution and Tween 80, 9:1, v/v. The dose of the red clover extract was selected to be 45 mg/kg/day in rats based on the human dose of 430 mg capsule per day (Red Clover Blossoms Capsules, Holland & Barrett Limited, Dublin). Red clover suspension was prepared by dispersing the capsule content in 0.2% Sodium CMC with 5% Tween 80. Rats were divided into four groups (n = 5, each). Group I: single oral dose of tamoxifen (10 mg/kg). Group II: 0.2% Sodium CMC with 5% Tween 80 was administered for 15 days. Group III: pretreatment with red clover (45 mg/kg/day) for 15 days. Group IV: pretreatment with red clover (45 mg/kg/day) for 15 days followed by administration of single oral dose of tamoxifen (10 mg/kg) 10 min after the last dose. Rats of all the groups were kept fasting for 12 h before the last dosing. Blood samples (approximately 0.25 mL) were collected from the retro-orbital plexus into heparinized microfuge tubes from group I and IV at 0.08, 0.50, 1, 3, 5, 7, 9, 24, 30 and 49 h post-dosing and plasma was harvested by centrifuging the blood at 15000 × g for 10 min and stored frozen at –70 ± 10 °C until bioanalysis. The rats of group II and III were anesthetized after 10 min of last dose with urethane at a dose of 1.5 g/kg and liver tissues were perfused with ice cold normal saline, excised and weighed. The little part of the liver was frozen in liquid nitrogen for mRNA extraction and remaining used for the preparation of the microsomes.

### LC-MS/MS analysis of tamoxifen and 4-hydroxytamoxifen

The generated study samples were analyzed for tamoxifen and 4-hydroxy tamoxifen by validated LC-MS/MS method published from our lab^7^,^40^,^41^. In brief, to 100 μL of plasma, 10 μL of IS (equivalent to 10 ng of centchroman) was added and mixed for 15 sec on a cyclomixer (Spinix Tarsons, Kolkata, India), followed by extraction with 2.0 mL of 5% v/v isopropanol in n-hexane. The mixture was vortexed for 3 min, followed by centrifugation for 5 min at 2000 × g on Sigma 3–16K centrifuge (Frankfurt, Germany). Organic phase was separated and evaporated. The residue was reconstituted with mobile phase and 10 μL of this solution was subjected to LC-MS/MS analysis. LC-MS/MS analysis was carried out using a HPLC system consisting of Shimadzu LC-20AD pumps and SIL-HTc auto sampler with a temperature controller and API 4000 QTrap MS/MS (Applied Biosystems, MDS Sciex Toronto, Canada). The separation was achieved on Supelco Discovery C18 column (4.6 × 50 mm, 5 μm) in isocratic mode with a mobile phase consisting of 0.01M ammonium acetate (pH4.5) and acetonitrile in a ratio of 10:90 (%v/v). The mass spectrometer was operated in positive ion mode and detection of the ions was performed in the multiple reaction monitoring (MRM) mode: monitoring transition of m/z 372.5 precursor ion [M+ H]^+^ to the m/z 72 product ion for tamoxifen, m/z 388.2 precursor ion [M+ H]^+^ to the m/z 72 product ion for 4-hydroxytamoxifen and m/z 458.5 precursor ion [M+ H]^+^ to the m/z 98.1 product ion for IS. The lower limit of quantification of the method was 0.2 ng/mL and linearity in the calibration curve standards was demonstrated up to an upper limit of 200 ng/mL. Study samples were analyzed along with quality control samples.

### Pharmacokinetic analysis

Plasma data was subjected to non-compartmental pharmacokinetics analysis using Phoenix WinNonlin (version 6.3, Pharsight Corporation, St. Louis, USA). The observed maximum plasma concentration (C_max_) and the time to reach the maximum plasma concentration (T_max_) were obtained by visual inspection of the experimental data. Area under the plasma concentration-time curve from time zero to the last quantifiable concentration (AUC_0-t_) was calculated using linear trapezoidal rule. The total area under the plasma concentration–time curve from time zero to time infinity (AUC_0-∞_) was calculated as the sum of AUC_0-t_ and C_last_/k_el_, where, C_last_ represents the last quantifiable concentration and K_el_ represents the terminal phase rate constant. The apparent elimination half-life (t_1/2_) was calculated as 0.693/k_el_ and the k_el_ was estimated by linear regression of the plasma concentrations in the log-linear terminal phase. The metabolic ratio (MR) was defined as the ratio of AUC value for 4-hydroxytamoxifen to that of tamoxifen. The relative bioavailability of tamoxifen was calculated as follows:


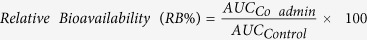


The mean metabolite ratio of tamoxifen was calculated using the formula:


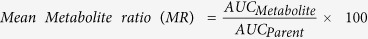


### Statistical analysis

The data is presented as a mean ± S.D. The pharmacokinetic parameters were compared using a student’s t-test test. A P-value of < 0.05 was considered significant.

### Determination of effect on expression of CYP isozymes

#### Total RNA extraction

Total RNA was extracted from 0.1–0.2 g of rat liver tissues by using TRIzol reagent as per manufacturer’s protocol. The quality and quantity of the isolated RNA was assessed using the 260/280 nm absorbance ratio (1.8–2.0 indicates a highly pure sample) using NanoDrop 2000c spectrophotometer (USA). Total RNA was stored at −80 °C until used.

#### Synthesis of cDNA and RT qPCR quantification

RNA was transcribed into cDNA in a 20 μl reaction using a High Capacity RNA to cDNA Reverse Transcriptase kit, analyzed, and amplified. 2x master mix was prepared using 2 μl 10 × Reverse Transcription buffer, 0.8 μl 25 × dinucleotide triphosphate (dNTP) mix (100mM), 2 μl 10x Reverse Transcription random primers (the primer sequences used were as given in Table 3) and 1 μl multiScribe Reverse Transcriptase. For a 20 μl reaction mixture under the following conditions: 5.8 μl master mix; 2 μl of 2 μg RNA sample; 12.2 μl nuclease free water. Following was the program for reverse transcriptase PCR using SureCycler 8800 (Agilent Technologies, USA): 25 °C for 10 mins; 37 °C for 120 mins; 85 °C for 5 mins; hold at 4 °C. The product obtained was diluted 4 × for real time PCR. Samples were seeded in triplicate in 96-well reaction plates (LightCycler 480). RT qPCR was performed on Light Cycler 480 II (Roche Diagnostics) with SYBR Green fluorescent label. Samples (10 μl final volume) contained the following: 2 × SYBR Premix Ex Taq 1 μl of each primer; 3 μl of cDNA sample and 5 μl of nuclease free water. Cycle threshold (Ct) values were used to calculate the amount of amplified PCR product relative to glyceraldehyde-3-phosphate dehydrogenase (GAPDH) as control (reference gene).

### Determination of microsomal CYP activities

The liver was homogenized in three volumes of ice-cold homogenization buffer consisting of 0.154 M KCl, 50 mM Tris-HCl, and 1 mM EDTA (pH 7.4). The homogenate was centrifuged at 10,000 g at 4 °C for 30 min. The supernatant was further centrifuged at 105,000 g at 4 °C for 60 min. The pellet was suspended in 50 mM Tris–HCl buffer containing 1mM EDTA (pH 7.4). Protein content was determined by Lowry method, using bovine serum albumin as a standard^42^. The concentration and activities of CYP were analyzed.

The prepared microsomes were tested for the activities of CYP 1a2, 2b2, 2c11, 2d4, 2e1and 3a2 by monitoring the index reactions phenacetin-O-deethylation, bupropion-hydroxylation, diclofenac 4′-hydroxylation, dextromethorphan O-demethylation, chlorzoxazone 6-hydroxylation and testosterone 6β–hydroxylation respectively. The rat liver microsomes (0.5 mg/mL) were incubated in 0.5 mL phosphate buffer (100 mM, pH 7.4) containing 10 mM MgCl_2_ at 37 °C in duplicate with a single isoform specific probe substrate concentration (25 μM phenacetin, 25 μM bupropion, 25 μM diclofenac, 5 μM dextromethorphan, 40 μM chlorzoxazone, 25 μM testosterone and 25 μM nifedipine). The reaction mixture was preincubated for 5 minutes at 37 °C. The reaction was started with the addition of NADPH (1.2 mM) and terminated by adding 0.5 mL of ice cold methanol containing internal standard. The reaction mixture was then vortexed for 5 minutes followed by centrifugation at 4,000 × g for 15 minutes resulting in removal of denatured proteins. An aliquot of 200 μL of supernatant was transferred to vial and analysed by high performance liquid chromatography (HPLC) using photo diode array (PDA) as detector. The liquid chromatographic system consisted of a Shimadzu LC-10AT*vp* pump, SIL-10AD*vp* autosampler, DGU-14A degasser, SPD- M10A*vp* photo diode array detector and CTO-10A*vp* column oven (Shimadzu Corporation, Kyoto, Japan). All the parameters of HPLC were controlled by Class VP software. The samples were run on Waters^®^ C18 column (250 mm × 4.5 mm, 5 μm). The rate of production of metabolite from the respective specific probe substrate were quantified using the ratio of the peak area of the metabolites to the peak area of internal standard with the help of appropriate standard curve generated using the authentic metabolite standard. Results were expressed as % activity of enzyme remaining with respect to the control liver microsomes.

### Determination of IC_50_ of red clover extract in human liver microsomes

The inhibitory effects of red clover extract, on CYP isoforms were determined in the concentration range in which they do not precipitate in the incubation mixture. The concentration range selected for extract was 6.25–250 μg/mL.

Incubations were performed at 37 °C containing human liver microsomes and a single isoform specific probe substrate concentration below the K_m_ values for their respective enzymes reported earlier from our lab (25 μM phenacetin (CYP1A2), 25 μM diclofenac (CYP2C9), 5 μM dextromethorphan (CYP2D6), 40 μM chlorzoxazone (CYP2E1), 25 μM testosterone (CYP3A4), and 25 μM bupropion (CYP2B6)) in the absence or presence of a range of concentrations of red clover extract. The reaction was initiated by addition of NADPH and terminated at the end of predetermined incubation period using suitable organic solvent. Processing and analysis of the samples were performed in the same way as mentioned above.

Positive controls (α-naphthoflavone (25 μM), sulfaphenazole (60 μM), quinidine (5 μM), 4-methyl pyrazole (80 μM), ketoconazole (1 μM) and ticlopidine (10 μM) were used as selective inhibitors of CYP1A2, CYP2C9, CYP2D6, CYP2E1, CYP3A4 and CYP2B6 activity, respectively) were also run in parallel under identical conditions in order to establish the reliability of the test system and hence results obtained. The rate of formation of the metabolite in the presence of isoform specific inhibitor was compared with that from controls in which inhibitor was replaced by equivalent volume of vehicle solvent. IC_50_ values were determined by analysing the plot of the logarithm of the inhibitor concentration versus percentage activity remaining after inhibition by using GraphPad Prism 5.0 software (GraphPad Inc., San Diego, CA, USA).

### Maintenance and treatment of HepG2 cells with red clover extract

HepG2 cells were procured from ATCC and were cultured according to ATCC protocols. HepG2 cells were cultured in Low Glucose DMEM (1 g/L glucose) supplemented with 10% low endotoxin FBS, 1X antibiotic-antimycotic solution at 37 °C, with 5% CO_2_ and 95% humidity. Cells were seeded at a concentration of 1 × 10^6^ cells/mL on a 60 × 15 mm cell culture sterile dishes (Corning, USA). After achieving 70–80% confluency the cells were treated with control (0.1% methanol) and different concentrations of red clover extract (125 and 250 μg/mL) for 72 hrs. The media was replaced every 24 h with fresh media containing red clover extract. The cells were lysed and processed for RT-qPCR.

#### Total mRNA extraction

Total RNA was extracted from HepG2 cells by using TRIzol reagent as per manufacturer’s protocol. The quality and quantity of the isolated RNA was assessed using the 260/280 nm absorbance ratio (1.8–2.0 indicates a highly pure sample) using NanoDrop 2000c spectrophotometer (USA). Total RNA was stored at −80 °C until used.

#### Synthesis of cDNA and RT qPCR quantification

RNA was transcribed into cDNA as described in previous section. Cycle threshold (Ct) values were used to calculate the amount of amplified PCR product relative to glyceraldehyde-3-phosphate dehydrogenase (GAPDH) as control (reference gene).

## Conclusion

The co-administration of red clover capsules as adjunct therapy along the tamoxifen has no affect the systemic exposure of the tamoxifen and its active metabolite 4-hydroxy tamoxifen. The expression and activities of CYP 1a1, 2b2, 2c11 and 3a4 were significantly altered by red clover treatment, indicating the potential for the interaction of red clover supplements with drugs which are substrates of these enzymes. The results obtained in the human liver preparations commensurate with those observed in the rats upon chronic treatment.

## Additional Information

**How to cite this article**: Rama Raju, K. S. *et al.* No effect on pharmacokinetics of tamoxifen and 4-hydroxytamoxifen by multiple doses of red clover capsule in rats. *Sci. Rep.* **5**, 16126; doi: 10.1038/srep16126 (2015).

## Figures and Tables

**Table 1:** Pharmacokinetic parameters of tamoxifen and 4-hydroxy tamoxifen following the oral administration of tamoxifen (10 mg/kg) with or without red clover (45 mg/kg/day) in rats (n = 5).

Parameters	Tamoxifen alone (Oral, 10 mg/kg)	Tamoxifen (Oral, 10 mg/kg) with red clover
Tamoxifen		
AUC_0-∞_ (hr*ng/mL)	1683.39 ± 71.77	1630.44 ± 17.74
C_max_ (ng/mL)	83.27 ± 13.03	75.63 ± 2.65
T_max_ (hr)	3.67 ± 1.15	3.00 ± 2.00
RB (%)	100	96.85
4-hydroxy tamoxifen		
AUC_0-∞_ (hr*ng/mL)	335.20 ± 42.66	286.84 ± 51.94
C_max_ (ng/mL)	11.34 ± 1.37	11.43 ± 2.71
T_max_ (hr)	5 ± 2.83	6.33 ± 1.15
RB (%)	100	85.57
MR	0.20	0.18

**Table 2:** IC_50_ values of Red clover extract for various CYP isoforms of human liver microsomes.

CYP isoform (Human)	Index Reaction	IC50 of Extract in human liver microsomes (μg/mL)
CYP1A2	Phenacetin O-deethylation	80.91
CYP3A4	Testosterone 6β-hydroxylation	89.52
CYP2B6	Bupropion hydroxylation	141.20
CYP2D6	Dextromethorphan O-demethylation	> 250
CYP2E1	Chlorzoxazone 6-hydroxylation	>250
CYP2C9	Diclofenac 4-hydroxylation	>250

**Table 3:** Primers of related genes for RT qPCR.

Isozyme	Forward Primers	Reverse Primers
rCYP1a1	CTACAACTCTGCCAGTCTCCAG	CCTCTCAACACCCAGAACACT
rCYP2b2	CATCTCATGCTGAGTCACTTCC	GACCATGGAGGGCTGATAAGT
rCYP2c11	GGAGACAGAGCTTTGGGAGA	CAATGATTGGGAGAGGTGTTG
rCYP2d4	CTCCAGACTTCTCGACTTGGTT	GGGTTTCTTTGGAAACACCTC
rCYP2e1	TGAGACCACCAGCACAACTC	CAATTTCTGGGTATTTCATGAGG
rCYP3a2	CCACTCACCCTGATATTCAGAAG	AGGTAGGAGGTGCCTTACTCG
rGADPH	TGGGAAGCTGGTCATCAAC	GCATCACCCCATTTGATGTT
hCYP1A2	TCCCACAGGAGAAGATTGTCA	CTCAGGCTTGGTCACAAGGT
hCYP2B6	AAAGCGGAGTGTGGAGGA	AAGGTGGGGTCCATGAGG
hCYP2C9	CGACAAGCACAACCCTGAG	TGCCAATCACACGTTCAATC
hCYP2D6	GCCCATCACCCAGATCCT	CAAGGTGGACACGGAGAAG
hCYP2E1	CAAGCCATTTTCCACAGGA	CAACAAAAGAAACAACTCCATGC
hCYP3A4	GATGGCTCTCATCCCAGACTT	AGTCCATGTGAATGGGTTCC
hGAPDH	AGCCACATCGCTCAGACAC	GCCCAATACGACCAAATCC

r- Represents rat isozymes.

h- Represents human HePG2 isozymes.
